# THE IMPACT OF GROUP-LED YOGA AND MINDFULNESS ON MENTAL AND PHYSICAL HEALTH IN SOUTH ASIAN WOMEN

**DOI:** 10.1093/geroni/igad104.3027

**Published:** 2023-12-21

**Authors:** Rozmin Jiwani, Mishel Malik, Sara Mithani, Sara Espinoza, Monica Serra

**Affiliations:** UT Health San Antonio, San Antonio, Texas, United States; UT Health San Antonio, San Antonio, Texas, United States; UT Health San Antonio, San Antonio, Texas, United States; Cedars-Sinai Medical Center, Los Angeles, California, United States; UT Health San Antonio, San Antonio, Texas, United States

## Abstract

One in five South Asians (people from India, Pakistan, and Bangladesh) living in the U.S. report experiencing a mental illness, with women reporting a three-fold greater risk of distress than men. South Asian women commonly interpret mental health symptoms as physical illnesses and resist seeking medical help. Yoga is a multifaceted, effective practice promoting well-being by integrating mind and body. This study aims to determine the feasibility and impact of a 12-week (Jan-April 2023) yoga and mindfulness intervention on mental and physical health outcomes in South Asian women. Thirty community-dwelling South Asian immigrant women, age ≥45 years were recruited to complete weekly 90-minute sessions of Ashtanga yoga including meditation and mindfulness practice. We are currently reporting baseline and 6-week data of mindfulness and physical activity self-reported questionnaires as the intervention is ongoing, but 12-week data will be available for the final presentation. At baseline, participants were 53±6 years old and a majority (80%) were overweight/obese with BMI ≥25kg/m2. At 6 weeks, twenty-eight (93%) participants continued with the study and twenty-five (83%) attended ≥4 of the 6 sessions. By 6 weeks, participants’ scores improved on Five Facet Mindfulness by 17% (p< 0.001) and Godin Leisure-Time Exercise by 15% (p>0.05) from baseline. These results suggest the preliminary feasibility, retention, and potential impact of the intervention on mental health outcomes in this population. Future directions include developing a large-scale, randomized lifestyle intervention trial to delay/manage mental health outcomes in South Asian women of all ages.

